# Patient-centered radiology reports with generative artificial intelligence: adding value to radiology reporting

**DOI:** 10.1038/s41598-024-63824-z

**Published:** 2024-06-08

**Authors:** Jiwoo Park, Kangrok Oh, Kyunghwa Han, Young Han Lee

**Affiliations:** 1 Department of Radiology, Research Institute of Radiological Science, and Center for Clinical Imaging Data Science (CCIDS), Yonsei University College of Medicine, 50-1 Yonsei-Ro, Seodaemun-Gu, Seoul, 03722 South Korea; 2 Institute for Innovation in Digital Healthcare, Yonsei University, Seoul, South Korea

**Keywords:** Large language model, Radiologic report, Patient-centered radiology, Artificial intelligence, Artificial hallucination, Medical imaging, Health care, Medical research

## Abstract

The purposes were to assess the efficacy of AI-generated radiology reports in terms of report summary, patient-friendliness, and recommendations and to evaluate the consistent performance of report quality and accuracy, contributing to the advancement of radiology workflow. Total 685 spine MRI reports were retrieved from our hospital database. AI-generated radiology reports were generated in three formats: (1) summary reports, (2) patient-friendly reports, and (3) recommendations. The occurrence of artificial hallucinations was evaluated in the AI-generated reports. Two radiologists conducted qualitative and quantitative assessments considering the original report as a standard reference. Two non-physician raters assessed their understanding of the content of original and patient-friendly reports using a 5-point Likert scale. The scoring of the AI-generated radiology reports were overall high average scores across all three formats. The average comprehension score for the original report was 2.71 ± 0.73, while the score for the patient-friendly reports significantly increased to 4.69 ± 0.48 (*p* < 0.001). There were 1.12% artificial hallucinations and 7.40% potentially harmful translations. In conclusion, the potential benefits of using generative AI assistants to generate these reports include improved report quality, greater efficiency in radiology workflow for producing summaries, patient-centered reports, and recommendations, and a move toward patient-centered radiology.

## Introduction

A radiologic report is generally the expression of medical images in words, and it is primarily focused on timely radiologic opinion with diagnostic accuracy for radiologists in their professional duties. While radiologic reports serve as a means of communication between radiologists and referring physicians, they are increasingly being provided directly to patients beyond the scope of inter-professional communication^1^. In addition, with the growing interest in healthcare among patients and the drive to enhance medical services toward patient-centered approach over the years, there has been an increased demand for radiologic reports that could be easily comprehensible to patients^2,3^.

However, radiologic reports are indeed intended for communication among medical experts, making it challenging for patients to understand the content of such reports with specialized and complex medical terminologies. One study reported that the average education level of patients is like a middle school student, and only 4% of the reports correspond to this level^4^. Nevertheless, it would be difficult, given the current medical system, to directly assign radiologists the task of providing patient-friendly reports, despite the reported benefits for patients to adhere to treatment plans and achieve better outcomes when they can comprehend their current radiological findings and their disease process^5,6^. As a result, there are alternative ongoing trials focused on enhancing the communication between attending physicians and their patients, such as patient portals and personal health records (PHRs) online^7–9^ to optimize patient engagement in treatment procedures.

Chat Generative Pre-trained Transformer (ChatGPT), an artificial intelligence (AI) chatbot released on November 30, 2022, is a technology based on the development of a large language model (LLM). In less than a year since its release, ChatGPT's language capability and its ability to surpass expectations in human-chatbot communication have received substantial attention across various fields, making it challenging to underestimate its influence^10,11^. Therefore, it is necessary to evaluate the impact of this model on healthcare from a medical perspective and, if possible, to seek ways to utilize it. Recently, medical studies, especially those concentrating on enhancing patient communication, have been conducted within a short period^12–15^. Notably, in a study comparing physician and AI chatbot responses, medical counseling by AI chatbot was evaluated as more preferred, of higher quality and empathy^12^. These results have significant implications for human doctors. Given ChatGPT’s friendly nature of conversation, it raises the question of whether we can expect patient-friendly interpretations of radiologic reports from such a helpful chatbot. Particularly, ChatGPT is known to be excellent, not only in composing text but also in summarizing^15–18^, for radiologists, while there are written reports available that derive key findings and conclusions from the imaging data. Hence, this study was initiated to anticipate a collaborate advantage arising from the combination of ChatGPT and radiology, paving the way for promising prospects.

However, when employing ChatGPT, it is essential to consistently address the ongoing issue of ‘artificial hallucination’, which refers to ChatGPT confidently presenting highly plausible yet glaringly incorrect statements in the form of self-assured responses, a notable weakness of LLMs in medical writing^14,19^. With its accessible web interface, patients have become potential users of this chatbot, where they can enter their medical reports at any time. Thus, the translated radiologic report by ChatGPT should be thoroughly evaluated by experienced radiologists to access the occurrence of artificial hallucination.

In this study, the objectives were to validate the effectiveness of AI-generated radiology reports in terms of report summary, patient-friendly reports, and recommendations and to evaluate the consistent performance of report quality and accuracy through the evaluation of artificial hallucinations.

## Methods

### Radiologic reports: set and generation

This retrospective study was approved by the institutional review board of Yonsei University Severance hospital (IRB 4-2023-0444), and the requirement for informed consent was waived. This study complied with the Declaration of Helsinki and the Health Insurance Portability and Accountability Act (HIPAA). We conducted this study using radiologic reports from magnetic resonance imaging (MRI), which is generally considered the most challenging imaging modality for patients in terms of comprehension. From December 1, 2022, to February 28, 2023, 685 spine MRI reports were retrieved from our hospital database, including 497 lumbar and 188 cervical spine MRIs of various disease groups at this tertiary referral center. All the MRI reports consisted entirely of English-language reports. Patient identification codes were removed for de-identification to ensure compliance with HIPAA regulations. The input reports included only the specified exam name, clinical information, findings, impressions, and recommendations, while the patient's name, age, gender, registration number, and PACS accession number were completely removed. The examples were shown in Supplementary Tables 6 and 7.

**Figure 1:**
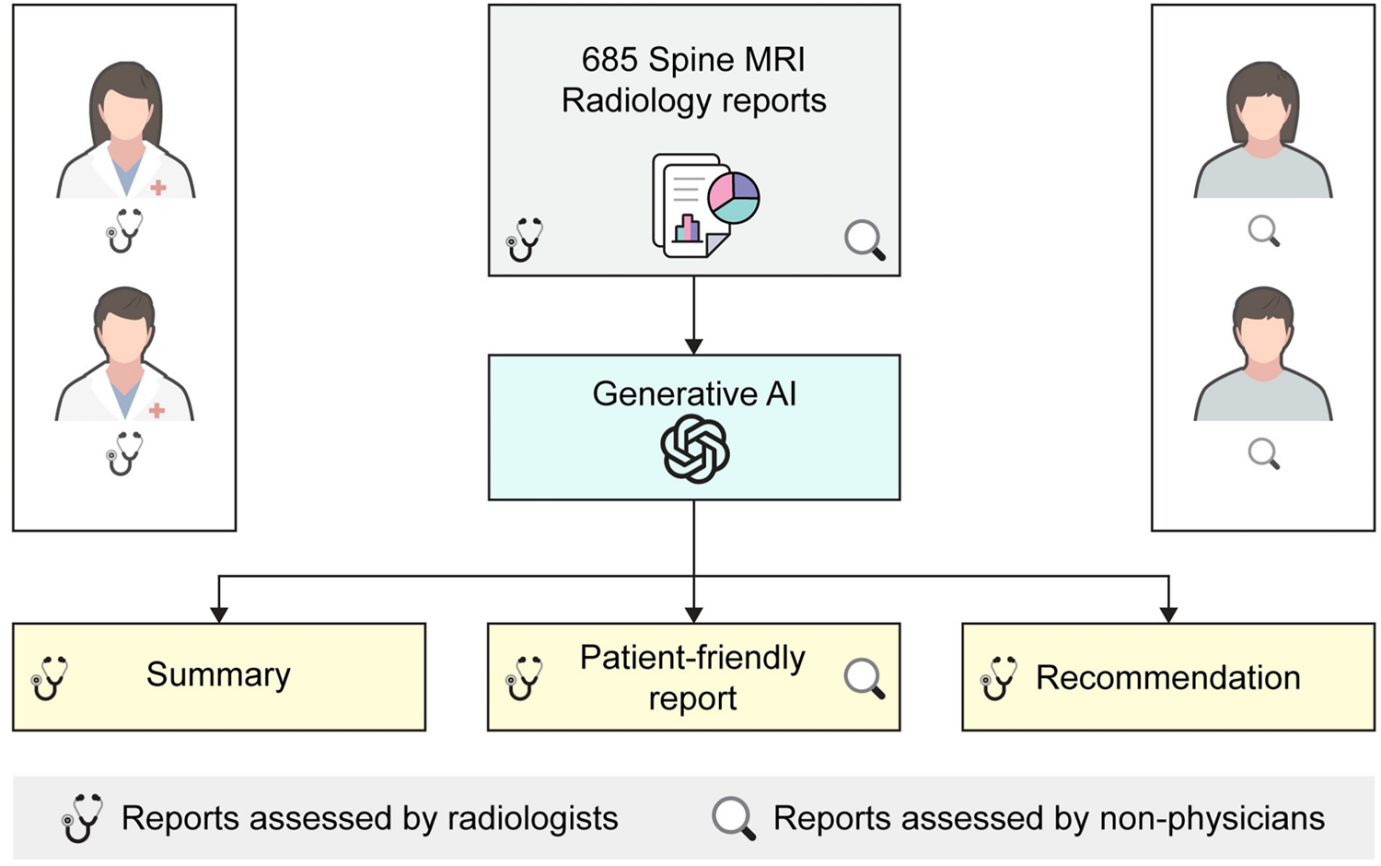
Flow diagram for development and evaluation datasets.

The dataset was developed from May 2, 2023, to May 22, 2023. All anonymized radiologic reports were inputted into the GPT-3.5-turbo model using Python's OpenAI application programming interface (API) to generate responses. During this process, we asked the GPT-3.5-turbo model to generate the following three formats of reports in English without any prior questions (i.e. zero-shot GPT) after presenting the original reports to the prompt: (1) Please make a summary. (2) Please make it easy for patients. (3) Please make a recommendation for the next step. And three formats were generated: (1) Summary, (2) Patient-friendly reports, and (3) Recommendations. The generated responses from the GPT-3.5-turbo model were evaluated and scored by four assessors, consisting of two radiologists and two non-physicians. The flow diagram for the development and evaluation of the dataset is summarized in Fig. 1.

### Evaluations of report generations by radiologists

Two fellowship-trained board-certified radiologists with 6–18 years of experience assessed the appropriateness and accuracy of three formats of reports by ChatGPT when considering the original report as a standard reference: (1) The quality of the summaries, (2) The compatibility of the patient-friendly reports, and (3) The concordance of the recommendations with those made by the radiologists. Additionally, the occurrences of artificial hallucinations were evaluated.

They rated the three formats of reports using a 5-point Likert scale^20,21^: 5, excellent; 4, good; 3, average; 2, below average; and 1, poor. The specific scoring criteria for the evaluation of three formats are detailed in Supplementary Tables 1, 2, and 3. The assessment guideline for the three formats were developed with a focus on the following aspects for each format. First, the quality of the summary was assessed based on whether it effectively captured all the important information in a concise format. Second, the compatibility of the patient-friendly report was evaluated based on its ability to explain the essential information to the patient in a more readable and easily understandable manner. Third, the concordance of the recommendations with those made by the radiologists was evaluated based on their consistency with the original report. Additionally, the word counts of the recommendation generated by ChatGPT and those of the original report were also compared.

Finally, the occurrence of artificial hallucinations, defined as the insertion of words or sentences that were entirely unrelated to the context or that create new pathologic conditions. If artificial hallucination was identified in the three formats, they were marked as “yes” along with the corresponding passage, while “no” was marked only when none were detected in any of the formats. The entire process was independently evaluated by two radiologists (J.P. and Y.H.L.). In the assessment of artificial hallucinations, two radiologists independently assessed the generated reports, and in cases where they had provided discordant evaluations, they underwent an additional consensus process to precisely define the scope of artificial hallucination. This step was needed because there had been no prior research on the subject, thus requiring an assessment process for the identified terms after individual review.

### Evaluations of report generations by non-physician raters

This study conducted an additional evaluation to verify the actual improvement in the comprehensibility of patient-friendly radiologic reports compared to the original reports. Two non-physicians, one a male in his thirties with a degree in computer science, and the other a female in her forties with a degree in statistics, both without any prior clinical medical experience, were provided radiologic reports that included both types of reports (patient-friendly and original). They read the reports and assessed their understanding of the content using a five-point Likert scale, where a score of 5 defined as 'Understood most of the content’ (an understanding level of over 90%); 4 as ‘Understood at least more than half’ (an understanding level of 50–90%); 3 as ‘Understood about half’ (an understanding level of approximately 50%); 2 as 'Understood less than half' (an understanding level of 10–50%); and 1 as ‘Hardly understood at all’ (understanding of less than 10%). The two non-physicians performed this process independently.

### Statistical analyses

Continuous variables were summarized with mean ± standard deviation. Categorical data were reported as the number of patients with percentages. The Kruskal–Wallis test was employed to compare the ratings among disease groups. The agreement between the two radiologists and between the two non-physicians was assessed using linearly weighted kappa statistics. The proportion of agreement with a 95% binomial exact confidence interval was presented, and kappa values of 0.41–0.60, 0.61–0.80, and 0.81–1.0 were considered to indicate moderate, substantial, and almost perfect inter-reader agreement, respectively^22^. Density plots were presented to compare the scores given by non-physicians for the original report with the patient-friendly report. The changes in score between the original report and the patient-friendly report were analyzed using Wilcoxon signed-rank test. *P* < 0.05 was considered to indicate statistical significance. All statistical analyses were performed in R software (version 4.3.0 (R Project for Statistical Computing)^23^.

## Results

### Clinical relevance statement

This study highlights the potential of AI-generated radiologic reports to improve report quality, enhance patient understanding, and facilitate patient-centered care. It also emphasizes the importance of meeting the needs and expectations of patients in radiologic practice.

### Clinical characteristics of the original MRI reports

The clinical characteristics of the 685 original MRI reports, finalized by a total of 11 fellowship-trained board-certified radiologists (7–25 years of experience in radiology), are summarized in Table 1. The mean age of patients who underwent spine MRI scans was 55 ± 22 years (mean ± standard deviation), including 355 men and 330 women. These patients presented with various lumbar spine disorders, and the frequencies within each disease category were as follows: degenerative disease (n = 446, 65.1%), tumors (n = 79, 11.5%), congenital disease (n = 63, 9.2%), trauma (n = 33, 4.8%), and infection/inflammation (n = 26, 3.8%), sequentially.

**Table 1:** The clinical characteristics of the original MRI reports.

	All	L-Spine	C-Spine
	(n = 685)	(n = 497)	(n = 188)
Age, mean ± SD	54.34 ± 22.52	57.81 ± 21.80	45.17 ± 21.85
Sex = M	355 (51.8)	254 (51.1)	101 (53.7)
Disease category			
No remarkable finding	14 (2.0)	5 (1.0)	9 (4.8)
Congenital disorder	63 (9.2)	40 (8.0)	23 (12.2)
Trauma	33 (4.8)	32 (6.4)	1 (0.5)
Degenerative disease	446 (65.1)	344 (69.2)	102 (54.3)
Infection/Inflammation	26 (3.8)	18 (3.6)	8 (4.3)
Tumor	79 (11.5)	55 (11.1)	24 (12.8)
Others*	24 (3.5)	3 (0.6)	21 (11.2)

Except where indicated, data are numbers of original MRI reports, with percentages in parentheses. SD, standard deviation.

*Others include Hirayama disease, arteriovenous fistula, epidural hemorrhage, subarachnoid cysts, bone marrow reconversion, syrinx, and syringomyelia.

### Qualitative and quantitative evaluation of the reports generated by ChatGPT: radiologist assessment

**Figure 2:**
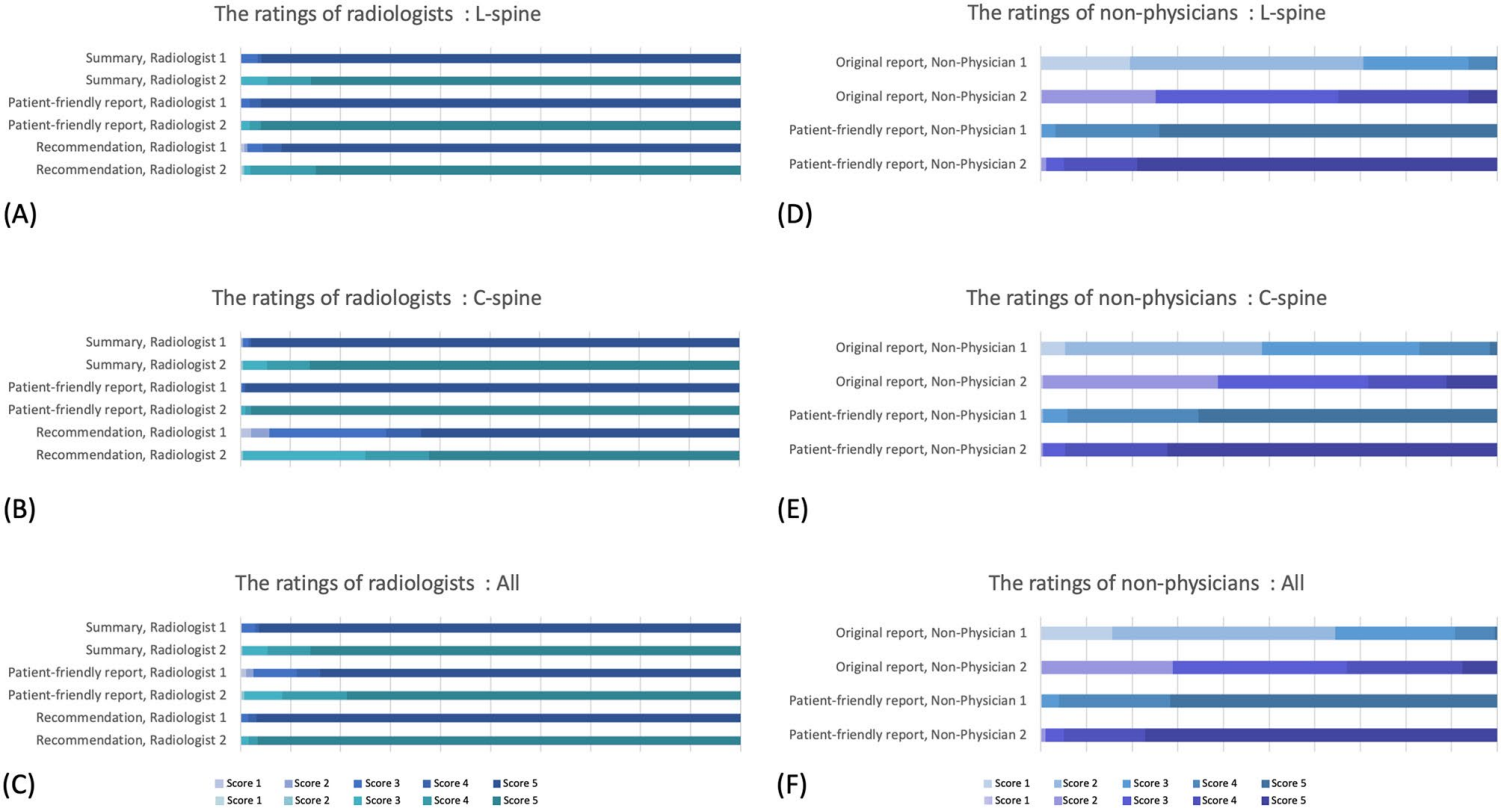
The ratings in L-spine and C-spine of non-physicians. The ratings of radiologists for summary, patient-friendly report, and recommendation are shown in L-spine (**A**), C-spine (**B**), and overall (**C**). The ratings of non-physicians for original report and patient-friendly report are shown in L-spine (**D**), C-spine (**E**), and overall (**F**).

Table 2 shows the evaluation of the three formats of reports generated by ChatGPT, as assessed by two radiologists independently (Fig. 2A–C). The average scores of the AI-generated radiologic reports were as follows: 4.86 ± 0.41 for the quality of the summary, 4.71 ± 0.60 for the compatibility of the patient-friendly reports, 4.94 ± 0.27 for the agreements of generative-AI recommendation with those made by the radiologists, and 4.84 ± 0.30 for the overall average scores across all three formats. And Supplement Table 4 presents the almost perfect inter-reader agreement between the two radiologists, ranging from 0.862 to 0.979. The representative examples for each score of respective reports and ratings according to the disease types are provided in Supplementary Tables 5, 6, 7, and 8.

**Table 2:** Summary statistics for the ratings in L-spine and C-spine of radiologists.

	All	L-Spine	C-Spine
Summary, reader 1	4.93 ± 0.38	4.92 ± 0.40	4.96 ± 0.31
Summary, reader 2	4.80 ± 0.54	4.80 ± 0.54	4.80 ± 0.54
Summary, reader-averaged	4.86 ± 0.41	4.86 ± 0.42	4.88 ± 0.36
Patient-friendly report, reader 1	4.69 ± 0.78	4.85 ± 0.57	4.27 ± 1.07
Patient-friendly report, reader 2	4.73 ± 0.54	4.83 ± 0.46	4.47 ± 0.63
Patient-friendly report, reader-averaged	4.71 ± 0.60	4.84 ± 0.44	4.37 ± 0.79
Recommendation, reader 1	4.95 ± 0.30	4.94 ± 0.34	4.98 ± 0.16
Recommendation, reader 2	4.94 ± 0.31	4.92 ± 0.33	4.97 ± 0.23
Recommendation, reader-averaged	4.94 ± 0.27	4.93 ± 0.30	4.98 ± 0.15
Overall average, reader 1	4.86 ± 0.35	4.90 ± 0.32	4.74 ± 0.39
Overall average, reader 2	4.82 ± 0.31	4.85 ± 0.30	4.75 ± 0.31
Overall average, reader-averaged	4.84 ± 0.30	4.88 ± 0.28	4.74 ± 0.31

Data are means ± standard deviation.

In addition, we also assessed the word counts of the reports, specifically focusing on the length of the recommendations. The average word count of the recommendations in the original reports was 3.00 ± 2.41, whereas the average word count for the recommendations generated by ChatGPT was 45.66 ± 15.97 (Table 3). And interestingly among the scores assigned to the three formats generated by ChatGPT and evaluated by radiologists, the response that provided a recommendation for the next step exhibited the highest quality score and the highest inter-reader agreement.

**Table 3:** The average word counts of the recommendations.

	All	L-Spine	C-Spine
Word counts, original report	3.00 ± 2.41	3.03 ± 2.51	2.86 ± 1.91
Word counts, ChatGPT	45.66 ± 15.97	46.59 ± 17.26	43.21 ± 11.61

### Comprehension verification of the patient-friendly reports generated by ChatGPT: non-physician assessment

**Figure 3:**
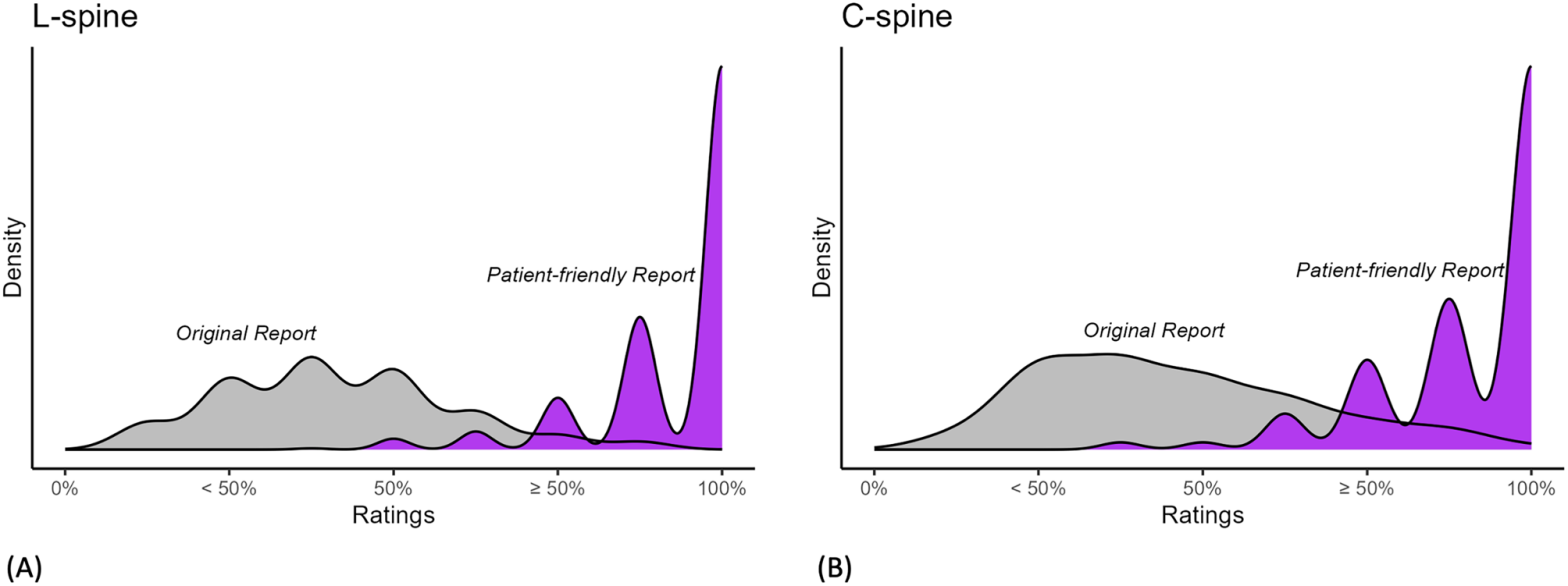
Distribution of average ratings for original reports and AI-generated patient-friendly reports. (**A**) L-spine MRI. (**B**) C-spine MRI.

In this study, two non-physicians assessed the comprehension of the original report and patient-friendly report, which verified an improvement in the level of understanding of the reports by the patients. Figure 3 shows the density plots of scores for each report providing the distribution of average scores given by the two non-physicians. Both non-physicians showed varying levels of comprehension of the original report. At the same time, it can be observed that the comprehension of the patient-friendly report was consistently higher, as indicated by the right-skewed distribution. Table 4 demonstrates the results, showing that the average comprehension score for the original report was 2.71 ± 0.73, while the score for the patient-friendly report increased to 4.69 ± 0.48. The rating distribution of non-physicians are shown in Fig. 2D–F. Significant improvements (*p* < 0.001) in comprehension were observed when comparing the patient-friendly report to the original report, although the agreement between the two non-physicians, particularly on the original reports, was not high, as shown in Supplementary Table 9. Additionally, ratings were obtained for each disease type (Supplementary Table 10), and there were also significant differences in comprehension depending on the disease type.

**Table 4:** Summary statistics for the ratings in L-spine and C-spine of non-physicians.

	All	L-Spine	C-Spine
Original report, reader 1	2.30 ± 0.85	2.16 ± 0.81	2.65 ± 0.86
Original report, reader 2	3.11 ± 0.92	3.16 ± 0.88	3.00 ± 1.01
Original report, reader-averaged	2.71 ± 0.73	2.66 ± 0.69	2.82 ± 0.81
Patient-friendly report, reader 1	4.67 ± 0.56	4.71 ± 0.53	4.59 ± 0.62
Patient-friendly report, reader 2	4.71 ± 0.59	4.73 ± 0.59	4.66 ± 0.59
Patient-friendly report, reader-averaged	4.69 ± 0.48	4.72 ± 0.46	4.63 ± 0.53
Improvement, reader 1	2.38 ± 0.93	2.54 ± 0.88	1.94 ± 0.93
Improvement, reader 2	1.60 ± 1.00	1.57 ± 0.99	1.66 ± 1.01
Improvement, reader-averaged	1.99 ± 0.80	2.06 ± 0.77	1.80 ± 0.84

Data are means ± standard deviation.

### Hallucination and potentially harmful expressions of the reports generated by ChatGPT: radiologist assessment

In this study, 23 cases (1.12%) of artificial hallucination with clinically significant implications were identified in 2,055 translated spine MRI reports by ChatGPT, with 16 cases (1.07%) in L-spine MRI and 7 cases (1.24%) in C-spine MRI, as agreed upon by two radiologists. Artificial hallucinations with clinically significant implications were observed only in the patient-friendly reports in 14 cases, while in 9 cases, they were observed concurrently in all three formats, including the summary and recommendation. Table 5 provides the representative expressions of artificial hallucinations repeatedly used in the AI-generated reports. 15 cases involved inaccurate explanations of medical terminology by adding information does not present in the original report to make it easier or overly simplified for patients. And most of the commonly used medical abbreviations were well understood, but there were 8 cases where totally misinterpretations occurred. In addition to the examples listed in Table 5, other translations that were slightly imprecise but generally did not cause issues in medical communication, were categorized separately as 'potentially harmful expressions,' (n = 152/2,055, 7.40%) not as artificial hallucinations. Included in these translated terms were as follows: “C-spine MRI” to “neck MRI,”; “contrast” to “dye,”; “disc bulging" to "wear and tear of the disc,"; “facet/uncovertebral joint or ligamentum flavum hypertrophy” to "joint or ligament swelling,"; “cord edema” to “cord swelling,” “intramedullary hematoma” to “bleeding inside your spinal cord,” respectively.

**Table 5:** Representative expressions of artificial hallucinations.

Original report	AI-generated report
4th lumbar vertebra – 1st sacral vertebra (L4-S1)	Between the fourth and ***first lumbar vertebrae***
Lumbar spine magnetic resonance imaging (MRI)	***The X-ray/CT*** of your lower back
Herniated lumbar disc (HLD)	***High-grade dysplasia***
***Disc protrusion***	Disc that is ***pressing/pushing out on a nerve***
***Disc extrusion***	
Schmorl’s node	A ***bone growth***
Hemangioma	

*The important statements are indicated in bold.

## Discussion

Effective communication is essential in patient-centered health care^24^, and radiologists ultimately communicate with patients based on their radiologic reports. The LLM has shown a significant impact on the field of radiology^11,25–28^. In this study, we aimed to investigate the potential of providing optimized communication to patients through patient-friendly radiologic reports using ChatGPT. Furthermore, we sought to assess the practical implications of such AI technology on patients and subsequent workflow in radiology. And this is why we employed ChatGPT, an LLM that has achieved historical record as the first successfully popularized AI model worldwide and has been widely adopted among the public.

The use of ChatGPT to generate radiologic reports that are more concise or easily understandable was an interesting and potentially valuable application of natural language processing technology. This study found that both formats of the transformed texts generated by ChatGPT demonstrated excellent quality and almost perfect agreement among readers. Summary of radiology report is an actively researched topic, and there are some reports have shown the excellent agreements of simplified reports as well^18,29^. These results demonstrate the potential of employing a reliable AI chatbot such as ChatGPT to translate radiologic reports that meet our established standards and expectations. The reason for requesting both a summary and a patient-friendly report from ChatGPT was that these texts serve different purposes, although they are closely related. The summary aimed to provide a concise report that includes all the essential information, even if medical terminology is used as it is^30^. On the other hand, the patient-friendly report focused on transforming the text to ensure that medical terminology is avoided and the content is presented in a way that patients can easily understand, prioritizing comprehension over the length of the text. Patient-friendly reports are designed to aid patient comprehension, while the summary could be used to communicate between radiologists and referring physicians, and it could be more useful, particularly for hospitals with non-structured original report formats. The LLM has demonstrated potential in converting unstructured data into structured data, indicating encouraging outcomes for standardization and data extraction^31,32^. The LLM based radiology report significantly enhances the readability and understandability of radiology reports^33,34^.

Furthermore, this study evaluated the difference in comprehension between the original and patient-friendly reports by the non-physicians, and although inter-rater agreement between the two non-physicians was not high, each of them demonstrated significant improvements. This suggests that regardless of the variation in individuals’ background knowledge, there was a substantial enhancement in understanding when reading the patient-friendly report compared to the original report, which had an average comprehension rate below 50%. So far, no study has evaluated the quality of radiologic reports generated by ChatGPT through assessments by radiologists and non-physicians, considering both accuracy and comprehensibility. This is one of the key strengths of our study, which has not been researched in previous studies. By providing both a summary report for medical professionals and a patient-friendly report for patients, ChatGPT has the potential to enhance communication, improve understanding, and ultimately contribute to better patient care in the field of radiology.

The recommendation system is a challenging application of natural language processing technology. This study found the highest scores in the use of AI-generated recommendations among the three formats in terms of the qualitative evaluation by radiologists and, unsurprisingly, showed a significant difference in length. It is common for our original reports, primarily focused on communication with healthcare professionals, to provide suggestions that consider clinical correlation for general-level results unless there is a specific emphasis on important recommendations. While respecting the physician’s expertise and the practical considerations of clinical situations, the report may be somewhat less cautious or empathetic when received by the patients. Similar to a study^12^, instead of a succinct one-word response from a human doctor, or even if it is a recommendation that pinpoints a single key point, people would perceive a comprehensive explanation by ChatGPT as superior, and it could be indicative of the compassionate attributes associated with ChatGPT.

Translation of radiologic reports using ChatGPT has shown many benefits, as previously discussed. However, the confident utilization of ChatGPT in the medical domain continues to raise questions. The presence of artificial hallucination in generative AI models, such as ChatGPT, represents is an important inherent issue when these models are applied in medical application. In this study, two radiologists thoroughly assessed the accuracy of the generated text. In the reports generated by ChatGPT, 1.12% of artificial hallucination was observed, and additionally, there was 7.40% inappropriate language that could potentially lead to misunderstandings in future communications. While the observed percentages of artificial hallucination may not be significantly high, they cannot be disregarded in medicine, which relies heavily on truthfulness, especially in non-healthcare professionals who could not aware of the potential issues they can pose. And ChatGPT is not fundamentally a model developed for healthcare purposes. Thus, radiologists are responsible for identifying and addressing artificial hallucinations moving forward to ameliorate the issues.

This study has a few limitations. First, this study was a single-center study using spine MRI reports. Thus, the consistency of our findings should be confirmed in the reports of other imaging modalities for other joints and organs as well. Second, we examined the potential of ChatGPT alone, and no other LLMs, in the unrestricted alterations of radiology reports. The consistency of findings should be verified in the recently introduced GPT4 with enhanced reasoning capability and in other LLMs. However, considering the aim of this paper, which focuses on whether general individuals without medical knowledge can understand medical reports through AI and the risks involved, the novelty or performance of the LLM is less critical than identifying which LLM is most widely known gratuitously available to the public. By using the ChatGPT, which can be deemed successful in its popularization among the public, the study aimed to identify and highlight the challenges associated with its usage, catering to the dual purpose of engaging medical professionals in the field of radiology and the public. Third, it should be noted that the evaluation in this study was conducted with two non-physician raters who lacked medical backgrounds. Although there are limitations in the number of evaluators in this study, we believe it lays the groundwork for future research exploring differences in comprehension and further investigations into AI-generated reports across a more diverse group of general individuals. The clinical significance highlighted by this study opens up the possibility of providing radiologic reports in a patient-friendly reports using LLMs immediately to real patients, and it can serve as a foundation for future research in this area. Fourth, the prompts used in this manuscript were determined without the assistance of prompt engineering expertise due to the study being in its early stages. Instead, they were made from clinical radiologic viewpoints. This could have influenced the effectiveness of the prompts and consequently the outcomes of the study. Using more crafted prompts by advanced prompt engineering is expected to yield better results^18,27^. Last limitation is the lack of direct validation of the content with patients. Alternatively, we included two adults of average knowledge level, one male and one female, to evaluate the comprehensibility of the reports, and matched them with two radiologists to assess the appropriateness and accuracy of the translated reports. Of course, the non-physician raters did not have professional medical knowledge. In future research, the evaluations from patients undergoing MRI scans or radiology report with a readability with specific level (e.g. eighth grade)^34,35^ is expected.

This study aims to evaluate the effectiveness of AI-generated radiology reports across various dimensions, including summaries, patient-friendly reports, and recommendations, thereby contributing to the enhancement of radiology workflows. The results consistently confirm their performance, maintaining accuracy. Furthermore, the study emphasizes the value of patient-centered radiology by improving comprehension through AI-generated patient-friendly reports, promoting better patient engagement and understanding of medical imaging results. While AI-generated reports may still have limitations in possibly incorporating false information, even in minor instances, their potential as a useful tool is convincingly demonstrated in this study. Rather than posing potential harm, they show promise as a valuable tool with proper oversight and correction by radiologists in the future.

### Supplementary Information

Supplementary Information.

## Data Availability

The datasets generated during and/or analyzed during the current study are available from the corresponding author on reasonable request.

## Abbreviations

AI: Artificial intelligence
API: Application programming interface
ChatGPT: Chat generative pre-trained transformer
LLM: Large language model
MRI: Magnetic resonance imaging
