# Supplementary material for: Patient-centered radiology reports with generative artificial intelligence: adding value to radiology reporting
Source: Sci Rep. 2024 Jun 8;14:13218. doi: 10.1038/s41598-024-63824-z (PMC11162416; doi:10.1038/s41598-024-63824-z)
Supplement: Supplementary file 1 — Supplementary Information. [file 41598_2024_63824_MOESM1_ESM.docx]

Supplement Table 1. The scoring criteria for the quality of the summary

| Score | Description |
| --- | --- |
| 5 point  ± excellent) | Fully includes the conclusion in the original report, and appropriately structured the overall content according to its importance. |
| 4 point  ± good) | Fully includes the conclusion in the original report, but also mentions too many findings, which may disrupt the overall structure. |
| 3 point  ± average) | Omits some parts of the conclusion in the original report ± e.g., neither the exact level of disc, nor the left or right sides, was mentioned.), but it does not significantly impact the communication. |
| 2 point  ± below average) | Omits significant parts of the conclusion in the original report or includes statements that may cause misunderstanding in further communication. |
| 1 point  ± poor) | Provides incorrect information not included in the original report. |

Supplement Table 2. The scoring criteria for the compatibility of the patient-friendly reports

| Score | Description |
| --- | --- |
| 5 point  ± excellent) | Includes the essential information that the patient needs to know using clear and understandable explanations, and the overall content is appropriate. |
| 4 point  ± good) | Includes the essential information that the patient needs to know using clear and understandable explanations, but also includes unnecessary statements. |
| 3 point  ± average) | Somewhat lacks in the explanation of medical terminology, but adequately provides the essential information that the patient needs to know. |
| 2 point  ± below average) | Omits the essential information that must be provided to the patient or includes statements that may cause misunderstanding in further communication. |
| 1 point  ± poor) | Provides incorrect information not included in the original report. |

Supplement Table 3. The scoring criteria for the concordance of the recommendations with those made by the radiologists

| Score | Description |
| --- | --- |
| 5 point  ± excellent) | Includes mentions that completely match the recommendation from the original report, along with appropriate additional information being suggested. |
| 4 point  ± good) | Includes mentions that completely match the recommendation from the original report. |
| 3 point  ± average) | Presents a similar direction with that of the recommendation from the original report. |
| 2 point  ± below average) | Contains some differences in the recommendation compared to the original report or includes inappropriate treatment suggestions that are not consistent with the severity of the disease. |
| 1 point  ± poor) | Presents recommendations that completely differ from the original report. |

Supplement Table 4. Interobserver agreement between the two radiologists

|  | L-Spine | C-Spine |
| --- | --- | --- |
| Summary | 0.885 (0.853, 0.911) | 0.872 (0.816, 0.916) |
| Patient-friendly Report | 0.862 (0.829, 0.891) | 0.883(0.828, 0.925) |
| Recommendation | 0.937 (0.912, 0.957) | 0.979 (0.946, 0.994) |

Note- Data are proportion of agreement. Data in parentheses are 95% CIs.

Supplement Table 5. Examples of each score for the quality of the summary

| Score | Original report | Chatbot response |
| --- | --- | --- |
| 5 point  Excellent | EXAM: L-spine MRI without contrast enhancement.  CLINICAL INFORMATION: Lower back pain and right lower leg radiating pain.    FINDINGS:  Lumbar scoliosis.  L1/2: Symmetric disc bulging.  L2/3: Symmetric disc bulging.  - sweeping of left L3 nerve sleeve.  L3/4: Asymmetric disc bulging.  L4/5: Right neural foraminal stenosis.  - right foraminal to extraforaminal disc protrusion  - right facet joint hypertrophy.  L5/S1: Mild right neural foraminal stenosis.  - right foraminal to extraforaminal disc protrusion.  Disc bulging at C5/6, C6/7, and T1/2.    CONCLUSION:  Right neural foraminal stenosis at L4/5 and L5/S1.    RECOMMENDATION:  Clinical correlation. | This report summarizes the findings from an L-spine MRI without contrast enhancement for a patient with lower back pain and ***right lower leg radiating pain.***  The MRI revealed lumbar scoliosis, disc bulging at multiple levels, and ***specific issues at L4/5 and L5/S1, including right neural foraminal stenosis with disc protrusion.***  Clinical correlation is recommended for further evaluation and determination of the best course of treatment. |
| 4 point  Good | EXAM: L-spine MRI with contrast enhancement.  CLINICAL INFORMATION: HCC ± HBV)  FINDINGS:  Bone marrow replacing lesion at L3, L4, and L5.   - associated pathologic fracture and extraosseous epidural extension with central canal compromise at L3, L4, and L5 levels. Suspicious bone marrow signal change with contrast enhancement at sacrum.    R/O Central spinal stenosis at C2/3 to C6/7.  R/O Right neural foraminal stenosis at C6/7.  R/O Left neural foraminal stenosis at C3/4 to C5/6.   CONCLUSION:  1. Multiple bone metastasis at L3, L4, and L5.   - pathologic fracture and extraosseous epidural extension with central canal compromise at L3, L4, and L5 levels.  2. R/O Bone metastasis at sacrum.    RECOMMENDATION:  Whole body bone scan. | The examination report describes the findings and interpretation of an MRI scan performed on a patient's lumbar spine with contrast enhancement.  The report indicates the presence of multiple bone metastases with pathologic fractures and epidural extension with central canal compromise at L3, L4, and L5 levels. ***There is a suspicious bone marrow signal change with contrast enhancement at the sacrum and a possibility of spinal stenosis at multiple levels.***  The report recommends further investigation with a whole-body bone scan to evaluate the extent of metastasis and determine the appropriate treatment plan. |
| 3 point  Average | EXAM: C-spine MRI without contrast enhancement. CLINICAL INFORMATION: Right arm numbness.   FINDINGS:  Fusion of C4-5. Focal T2 hyperintense lesion in spinal cord at fused C4-5 level.  Right neural foraminal stenosis at C5/6 and bilateral neural foraminal stenosis at C7/T1.  - due to uncovertebral joint hypertrophy.  Osteochondrosis with decreased intervertebral disc space at C5/6, C6/7, and C7/T1.   Disc bulging at L3/4 and L4/5.  CONCLUSION:  Focal T2 hyperintense lesion in spinal cord at fused C4-5 level, indeterminate clinical significance. Right neural foraminal stenosis at C5/6 and bilateral neural foraminal stenosis at C7/T1 by uncovertebral joint hypertrophy.  RECOMMENDATION:  Clinical correlation. | The C-spine MRI without contrast enhancement showed fusion of C4-5, a focal T2 hyperintense lesion in the spinal cord at the fused level with uncertain clinical significance, and ***neural foraminal stenosis at C5/6 and C7/T1 due to uncovertebral joint hypertrophy.*** The report also noted osteochondrosis with decreased intervertebral disc space at multiple levels, and disc bulging at L3/4 and L4/5.  Clinical correlation is recommended. |
| 2 point  Below  Average | EXAM: L-spine MRI without contrast enhancement.  CLINICAL INFORMATION: s/p Endoscopic discectomy, L3/4 ± 2022-12-27), s/p Disc operation, L4/5, Lt ± outside, 16 years ago)  FINDINGS:  In reference to 2022.12.26 L-spine CT,  s/p Endoscopic discectomy, L3/4.   - improved central canal stenosis, with minimal residual central zone disc protrusion.  Left subarticular zone disc protrusion at L4/5.  Modic change ± Type 2) with osteochondrosis and disc space narrowing, L4/5.  No spinal cord signal change.    CONCLUSION:  Improved central canal stenosis with minimal residual disc protrusion, L3/4.  R/O Left lateral canal stenosis, L4/5.    RECOMMENDATION:  Clinical correlation. | The MRI of the lumbar spine was performed on a patient with ***a history of endoscopic discectomy at L3/4 and disc operation at L4/5, 16 years ago.***  The MRI shows an improvement in central canal stenosis with minimal residual disc protrusion at L3/4. However, there is a left subarticular zone disc protrusion at L4/5 ***causing left lateral canal stenosis,*** and Modic change ± Type 2) with osteochondrosis and disc space narrowing at L4/5.  ***The findings suggest a postoperative status*** and require further clinical correlation. |
| 1 point  Poor | EXAM: C-spine MRI with whole sagittal T2-weighted images. CLINICAL INFORMATION: Neck pain with right radiculopathy for 3 months.  FINDINGS: C3/4: Right neural foraminal stenosis, right central disc protrusion, and right uncovertebral joint hypertrophy. C4/5: Right central disc protrusion. C5/6: Left central disc protrusion. C6/7: Central disc protrusion.  Disc bulging at T12/L1, L1/2, L2/3, L4/5 and L5/S1. Perineural cyst at left S1 nerve sleeve at S1 level. Degenerative endplate changes in L4/5 and L5/S1.  CONCLUSION: Right neural foraminal stenosis at C3/4. Disc protrusions at C3/4 through C6/7.  RECOMMENDATION:  Clinical correlation. | The C-spine MRI without contrast enhancement showed ***right neural foraminal stenosis, disc protrusions, and degenerative changes in the lower spine.***  The recommendation is for clinical correlation. |

* The important statements assigned to each score are indicated in bold.

Supplement Table 6. Examples of each score for the compatibility of the patient-friendly reports

| Score | Original report | Chatbot response |
| --- | --- | --- |
| 5 point  Excellent | EXAM: L-spine MRI with whole-sagittal T2 image  CLINICAL INFORMATION: Back pain and right radiculopathy.   FINDINGS: T12/L1: Right central to subarticular disc protrusion abutting right L1 nerve root. No significant central canal nor neural foraminal stenosis. No abnormality in spinal cord.   CONCLUSION:  HLD, right central to subarticular protrusion, T12/L1.   RECOMMENDATION:  Clinical correlation. | You have back pain and right radiculopathy, ***which means you have pain, numbness, or weakness in your right leg.*** Imaging studies show a disc protrusion in your spine at the T12/L1 level, ***which is putting pressure on the nerve that goes to your right leg.*** There are no significant blockages or abnormalities in your spinal cord. |
| 4 point  Good | EXAM: L-spine MRI with whole-sagittal T2 image    FINDINGS:  L2/3: Disc bulging.  L3/4: Moderate central canal stenosis with disc bulging, ligamentum flavum thickening and facet joint hypertrophy.  No significant neural foraminal stenosis.  Ossification of posterior longitudinal ligament ± OPLL) at T1-2-3 level, without significant cord signal change.    CONCLUSION:  Moderate central canal stenosis at L3/4.  OPLL at T1-2-3 level.    RECOMMENDATION:  Clinical correlation. | You had an MRI of your lower spine, which showed that the discs between the vertebrae at the level L2/3 are bulging slightly. At the level L3/4, there is moderate narrowing of the space that the spinal cord goes through, along with bulging of the disc, thickening of a ligament, and swelling of the facet joints. There is no significant narrowing of the holes where the nerves come out. Also, there is a condition called "ossification of posterior longitudinal ligament" ± OPLL) which means that a ligament in your spine has hardened and become more bony at the levels T1-2-3, but this is not causing any problems for your spinal cord.  Overall, the MRI shows that you have moderate central canal stenosis at L3/4 and OPLL at T1-2-3 level. Your doctor will talk to you about what this means and what you should do next. |
| 3 point Average | EXAM: C-spine MRI with contrast enhancement.  CLINICAL INFORMATION: Submandibular gland adenocarcinoma.  FINDINGS:  Compared with previous C-spin MRI taken on 2022.7.7,   s/p Anterior cervical discectomy and fusion ± ACDF) in C5-6. Right paravertebral enhancing soft tissue lesions at C5 and C6 levels. - increase in size of enhancing lesions. - extension to right C5/6 and C6/7 neural foramina.  - abutting right C6 nerve root. - increase in size of epidural extension. No significant change of bone marrow replacing mass in C7. Both neural foraminal stenosis, disc bulging and both uncovertebral joint hypertrophy at C6/7.  s/p Posterior lumbar interbody fusion at L5-S1.  No change of central canal stenosis at L4/5. Disc bulging at L3/4 and L4/5.  CONCLUSION: Paravertebral metastatic soft tissue lesion in C5 and C6 levels. - extension to right C5/6 and C6/7 neural foramina. - abutting right C6 nerve root. - progression of epidural extension.  No change of metastatic bone lesion at C7.  RECOMMENDATION:  Clinical correlation. | The C-spine MRI with contrast enhancement shows that the cancer has grown in the soft tissue at the right side of your neck, specifically at the C5 and C6 levels. The cancer has also spread to the right C5/6 and C6/7 neural foramina, which are the openings where the nerves exit the spinal cord. ***This has caused narrowing of the right neural foramen*** and an increase in the size of the cancerous tissue. The report suggests that the cancer have invaded the right C6 nerve root. There is no significant change in the cancerous bone lesion at C7.  Your doctor will discuss the findings with you and recommend the next steps. |
| 2 point  Below  Average | EXAM: C-spine MRI with whole sagittal T2 image.   FINDINGS:  Loss of normal cervical lordosis.  Mild volume of central disc protrusion at C3/4.  Disc bulging and uncovertebral joint hypertrophy with left neural foraminal stenosis at C5/6 and C6/7.  No remarkable spinal cord signal change.  Multilevel disc protrusion/ bulging at thoracolumbar spines.  Bilateral neural foraminal stenosis at L5/S1.   A probable hemangioma at T3 vertebral body showing T1 and T2 high signal intensity.  CONCLUSION:  Left neural foraminal stenosis at C5/6 and C6/7.   RECOMMENDATION:  Clinical correlation. | The MRI showed that the normal curve in your neck is lost, and there is a small bulge in one of your discs. There is also a bulge in some of the discs in your upper and lower back, which is causing narrowing of the spaces where your nerves exit. There is a possible growth in one of your vertebrae, but we need to do more tests to confirm. We need to talk to you and examine you to see how these findings relate to your symptoms. |
| 1 point  Poor | EXAM: L-spine MRI with contrast enhancement.  CLINICAL INFORMATION: HCC.   FINDINGS:  Compared with previous MRI taken on 2022-11-25,  Slightly increased size of bone metastasis in sacral bone at S1 and S2 levels.  - Epidural extension at S1 and S2 levels.  - Abutment with left S1 nerve root and encasement of left S2 and S3 nerve root.  No significant leptomeningeal enhancement.  Disc bulging at C3/4 through T1/2 and L3/4 through L5/S1.  Asymmetric disc bulging and bilateral facet joint hypertrophy at L4/5.  Left central disc extrusion with annular fissure at L5/S1.    Limited evaluation of C2 and thyroid lesion due to lack of appropriate sequences at cervical level.    CONCLUSION:  Increased size of bone metastasis in sacrum at S1 and S2 levels.  - Epidural extension at S1 and S2 levels.  - R/O Invasion to left S1, S2 and S3 nerve roots.    RECOMMENDATION:  Clinical correlation. | The imaging test showed that the cancer has slightly grown in your sacrum bone, Furt some of it pushing on the nerves in your lower back. There is also some pressure on the nerves in your neck. The test also found some problems with the discs and joints in your lower back. We need more information and tests to know exactly what is going on. |

* The important statements assigned to each score are indicated in bold.

Supplement Table 7. Examples of each score for the concordance of the recommendations with those made by the radiologists

| Score | Original report | Chatbot response |
| --- | --- | --- |
| 5 point  Excellent | EXAM: L-spine MRI with contrast enhancement. CLINICAL INFORMATION: Back pain, CRP 63.0mg/L ± normal 0-8mg/L).  FINDINGS : Endplate erosion with contrast enhancement of L5/S1. - Fluid signal on L5/S1 disk. - Combined adjacent bone marrow and paraspinal soft tissue enhancement. No significant prevertebral and epidural abscess formation. L3/4: Symmetric disc bulging. L4/5: Right central disc protrusion. L5/S1: Symmetric bulging disc with mild bilateral spinal stenosis. T2 hyperintense lesion in T10 and preserved trabeculation, probably vertebral hemangioma.  CONCLUSION: Spondylodiscitis at L5-S1. HLD, L4/5.  RECOMMENDATION:  Follow-up with contrast enhanced L-spine MRI. | The patient should discuss their symptoms and medical history with their doctor to determine the best course of treatment for their spondylodiscitis and HLD. ***A follow-up MRI with contrast enhancement should be scheduled to monitor any changes or progression of the conditions.*** The patient may also benefit from physical therapy, pain management, and/or antibiotics if an infection is present. |
| 4 point  Good | EXAM: L-spine MRI with whole sagittal T2 image.  CLINICAL INFORMATION: Lung cancer, gastric cancer.   FINDINGS:  Suboptimal image quality due to dielectric artifact. No definite evidence of bone marrow replacing lesion.  No definite evidence of leptomeningeal metastasis.  Recent compression fracture with upper endplate depression at L2.  Retrolisthesis at L3/4.  Degenerative spondylolisthesis with disc bulging at L5/S1.   - causing central canal narrowing.  Subcutaneous edema along the lumbosacral spine.  Disc bulging/ protrusion at C5/6.   CONCLUSION:  Limited evaluation due to dielectric effects. 1. No definite evidence of metastasis in visualized skeleton. 2. Recent compression fracture at L2, more likely benign.   RECOMMENDATION:  Follow-up with 1.5T MRI. Correlation with other imaging study including whole body bone scan ± WBBS). | Based on the limited evaluation due to dielectric effects, it is recommended that the patient undergoes clinical correlation with ***a follow-up 1.5T MRI and correlation with other imaging studies, including a whole-body bone scan.*** This will provide a more comprehensive understanding of the patient's condition and guide further treatment decisions. |
| 3 point Average | EXAM: L-spine MRI without contrast enhancement. CLINICAL INFORMATION: Lower back pain.  FINDINGS:  L3/4: Disc bulging, facet joint hypertrophy and ligament flavum thickening. L4/5: Right neural foraminal stenosis by bulging disc, ligament flavum thickening and facet joint hypertrophy. Degenerative endplate change on L4 and L5 ± Modic type 1 and 3). Paravertebral muscle atrophy. R/O Central spinal stenosis at C5/6 and C6/7. R/O Bilateral neural foraminal stenosis at C5/6 and C6/7. Multiple renal cysts, both.  CONCLUSION:  Right neural foraminal stenosis by bulging disc at L4/5. Degenerative endplate change on L4 and L5 ± Modic type 1 and 3).  RECOMMENDATION:  Clinical correlation and C-spine MRI including axial scan if clinically indicated. | The patient should follow up with their doctor to discuss the results of the MRI and any further testing or treatment that may be necessary. The doctor may recommend physical therapy, medication, or other interventions to manage the lower back pain and address any potential issues in the neck. The patient should also discuss the renal cysts with their doctor to determine if any further evaluation or treatment is necessary. |
| 2 point  Below  Average | EXAM: L-spine MRI without contrast enhancement.  CLINICAL INFORMATION: Back pain with left radiculopathy.  FINDINGS:  L4/5: Central zone disc protrusion, with severe central canal stenosis.  No spinal cord signal change or bone marrow signal abnormality.  No neural foraminal stenosis.    CONCLUSION:  Severe central canal stenosis, L4/5.    RECOMMENDATION:  Clinical correlation. | Based on the imaging results, it is recommended that the patient undergo further clinical evaluation ***to determine the best course of treatment*** including physical therapy, medication, or surgery. The patient should be closely monitored for any changes in symptoms and follow-up imaging may be necessary. |

* The important statements assigned to each score are indicated in bold.

* There were no instances where the concordance of the recommendations with those made by the radiologists was rated with one point.

Supplement Table 8. Summary statistics for the ratings of radiologists according to the disease types

|  | 0 | 1 | 2 | 3 | 4 | 5 | 6 | P |
| --- | --- | --- | --- | --- | --- | --- | --- | --- |
| n | 14 | 63 | 33 | 446 | 26 | 79 | 24 |  |
| Summary, reader 1 | 5.00 ± 0.00 | 4.98 ± 0.13 | 5.00 ± 0.00 | 4.94 ± 0.34 | 4.77 ± 0.65 | 4.82 ± 0.66 | 5.00 ± 0.00 | 0.018 |
| Summary, reader 2 | 4.86 ± 0.36 | 4.90 ± 0.35 | 4.91 ± 0.29 | 4.78 ± 0.56 | 4.69 ± 0.74 | 4.82 ± 0.62 | 4.92 ± 0.28 | 0.319 |
| Summary, reader-averaged | 4.93 ± 0.18 | 4.94 ± 0.20 | 4.95 ± 0.15 | 4.86 ± 0.38 | 4.73 ± 0.67 | 4.81 ± 0.62 | 4.96 ± 0.14 | 0.121 |
| Patient-friendly report, reader 1 | 4.71 ± 0.73 | 4.83 ± 0.64 | 4.88 ± 0.48 | 4.68 ± 0.78 | 4.62 ± 0.94 | 4.73 ± 0.76 | 4.25 ± 1.26 | 0.062 |
| Patient-friendly report, reader 2 | 4.79 ± 0.43 | 4.92 ± 0.27 | 4.79 ± 0.60 | 4.70 ± 0.53 | 4.77 ± 0.51 | 4.78 ± 0.60 | 4.50 ± 0.72 | 0.022 |
| Patient-friendly report, reader-averaged | 4.75 ± 0.55 | 4.87 ± 0.35 | 4.83 ± 0.48 | 4.69 ± 0.59 | 4.69 ± 0.63 | 4.75 ± 0.66 | 4.38 ± 0.95 | 0.024 |
| Recommendation, reader 1 | 5.00 ± 0.00 | 4.98 ± 0.13 | 4.85 ± 0.51 | 4.94 ± 0.33 | 5.00 ± 0.00 | 4.96 ± 0.25 | 5.00 ± 0.00 | 0.36 |
| Recommendation, reader 2 | 5.00 ± 0.00 | 4.92 ± 0.37 | 4.82 ± 0.58 | 4.94 ± 0.30 | 4.92 ± 0.27 | 4.97 ± 0.16 | 5.00 ± 0.00 | 0.25 |
| Recommendation, reader-averaged | 5.00 ± 0.00 | 4.95 ± 0.23 | 4.83 ± 0.51 | 4.94 ± 0.28 | 4.96 ± 0.14 | 4.97 ± 0.17 | 5.00 ± 0.00 | 0.236 |

Note- Data are means ± standard deviation. 0, no remarkable finding; 1, congenital disease; 2, trauma; 3, degenerative disease; 4, infection/ inflammation; 5, tumor; 6, others*.

* Others include Hirayama disease, arteriovenous fistula, epidural hemorrhage, subarachnoid cysts, bone marrow reconversion, syrinx, and syringomyelia.

Supplement Table 9. Interobserver agreement between the two non-physicians

|  | L-Spine | C-Spine |
| --- | --- | --- |
| Original Report | 0.254 (0.216, 0.294) | 0.404 (0.333, 0.478) |
| Patient-friendly Report | 0.718 (0.677, 0.757) | 0.697 (0.626, 0.762) |

Note- Data are proportion of agreement. Data in parentheses are 95% CIs.

Supplement Table 10. Summary statistics for the ratings of non-physicians according to the disease types

|  | 0 | 1 | 2 | 3 | 4 | 5 | 6 | P |
| --- | --- | --- | --- | --- | --- | --- | --- | --- |
| n | 14 | 63 | 33 | 446 | 26 | 79 | 24 |  |
| Original report, reader 1 | 2.86 ± 1.17 | 2.00 ± 0.80 | 2.21 ± 0.93 | 2.37 ± 0.82 | 1.96 ± 0.92 | 2.08 ± 0.84 | 2.62 ± 0.82 | <0.001 |
| Original report, reader 2 | 3.71 ± 1.27 | 2.35 ± 0.57 | 3.27 ± 0.84 | 3.16 ± 0.88 | 3.27 ± 1.04 | 3.24 ± 0.94 | 3.12 ± 1.12 | <0.001 |
| Original report, reader-averaged | 3.29 ± 1.07 | 2.17 ± 0.55 | 2.74 ± 0.74 | 2.76 ± 0.70 | 2.62 ± 0.79 | 2.66 ± 0.73 | 2.88 ± 0.82 | <0.001 |
| Patient-friendly report, reader 1 | 4.71 ± 0.47 | 4.33 ± 0.70 | 4.61 ± 0.66 | 4.78 ± 0.48 | 4.42 ± 0.58 | 4.52 ± 0.60 | 4.38 ± 0.65 | <0.001 |
| Patient-friendly report, reader 2 | 4.86 ± 0.36 | 4.14 ± 0.90 | 4.85 ± 0.44 | 4.79 ± 0.49 | 4.65 ± 0.69 | 4.63 ± 0.64 | 4.75 ± 0.53 | <0.001 |
| Patient-friendly report, reader-averaged | 4.79 ± 0.32 | 4.24 ± 0.69 | 4.73 ± 0.49 | 4.79 ± 0.40 | 4.54 ± 0.45 | 4.58 ± 0.51 | 4.56 ± 0.45 | <0.001 |
| Improvement, reader 1 | 1.86 ± 1.35 | 2.33 ± 1.06 | 2.39 ± 0.90 | 2.42 ± 0.88 | 2.46 ± 1.10 | 2.44 ± 0.94 | 1.75 ± 0.68 | 0.009 |
| Improvement, reader 2 | 1.14 ± 1.29 | 1.79 ± 0.95 | 1.58 ± 0.90 | 1.63 ± 0.95 | 1.38 ± 1.27 | 1.39 ± 1.13 | 1.62 ± 1.17 | 0.11 |
| Improvement, reader-averaged | 1.50 ± 1.14 | 2.06 ± 0.86 | 1.98 ± 0.78 | 2.02 ± 0.75 | 1.92 ± 1.03 | 1.92 ± 0.85 | 1.69 ± 0.78 | 0.091 |

Note- Data are means ± standard deviation. 0, no remarkable finding; 1, congenital disease; 2, trauma; 3, degenerative disease; 4, infection/ inflammation; 5, tumor; 6, others*.

* Others include Hirayama disease, arteriovenous fistula, epidural hemorrhage, subarachnoid cysts, bone marrow reconversion, syrinx, and syringomyelia
